# Synthesis and Evaluation of a Sodium Alginate-4-Aminosalicylic Acid Based Microporous Hydrogel for Potential Viscosupplementation for Joint Injuries and Arthritis-Induced Conditions

**DOI:** 10.3390/md15080257

**Published:** 2017-08-16

**Authors:** Dharmesh R. Chejara, Mostafa Mabrouk, Pradeep Kumar, Yahya E. Choonara, Pierre P. D. Kondiah, Ravindra V. Badhe, Lisa C. du Toit, Divya Bijukumar, Viness Pillay

**Affiliations:** 1 Wits Advanced Drug Delivery Platform Research Unit, Department of Pharmacy and Pharmacology, Faculty of Health Sciences, School of Therapeutics Sciences, University of the Witwatersrand, Johannesburg, 7 York Road, Parktown 2193, South Africa; dharmeshchejara@gmail.com (D.R.C.); mostafamabrouk.nrc@gmail.com (M.M.); pradeep.kumar@wits.ac.za (P.K.); yahya.choonara@wits.ac.za (Y.E.C.); pierre.kondiah@wits.ac.za (P.P.D.K.); badheravi2@gmail.com (R.V.B.); lisa.dutoit@wits.ac.za (L.C.d.T.); divyaraniv@gmail.com (D.B.); 2 Refractories, Ceramics and Building Materials Department, National Research Centre, 33El Bohouth St. (former El-Tahrir St.), Dokk P.O.12622i, Giza, Egypt

**Keywords:** hydrogel, sodium alginate, 4-aminosalicylic acid (4-ASA), viscosupplementation, arthritis

## Abstract

A microporous hydrogel was developed using sodium alginate (alg) and 4-aminosalicylic acid (4-ASA). The synthesized hydrogel was characterized using various analytical techniques such as Fourier transform infrared spectroscopy (FTIR), Carbon-13 nuclear magnetic resonance (^13^C-NMR), X-ray powder diffraction (XRD), scanning electron microscopy (SEM), and differential scanning calorimetry (DSC). Additonal carboxyl and hydroxyl functional groups of 4-ASA provided significant lubrication and stress-triggered sol-gel transition to the conjugated hydrogel. In addition, cytotoxicity analysis was undertaken on the conjugated hydrogel using human dermal fibroblast-adult (HDFa) cells, displaying non-toxic characteristics. Drug release profiles displaying 49.6% in the first 8 h and 97.5% within 72 h, similar to the native polymer (42.8% in first 8 h and 90.1% within 72 h). Under applied external stimuli, the modified hydrogel displayed significant gelling properties and structure deformation/recovery behaviour, confirmed using rheological evaluation (viscosity and thixotropic area of 8095.3 mPas and 26.23%, respectively). The modified hydrogel, thus, offers great possibility for designing smart synovial fluids as a biomimetic aqueous lubricant for joint-related injuries and arthritis-induced conditions. In addtion, the combination of thixotropy, non-toxicity, and drug release capabilities enables potential viscosupplementation for clinical application.

## 1. Introduction

In recent years, the demand for advancement of artificial orthopedic implants for bone and joint disorders has significantly increased. Researchers have shown the advantages of using natural polymer-based materials, due to their biodegradable and biocompatible application [1,2,3,4]. Natural polysaccharides have been acknowledged as attractive candidates for such applications, due to their abundant economical availability and modification for tailor-made properties [5,6,7]. Thus, the use of natural polymers, such as sodium alginate carrageenan, agarose, chitosan, and cellulose, have attractive properties for applications in response to stress and strain parameters, ideal for viscosupplementation therapy. Chemically-modified polymers are frequently used in pharmaceuticals, healthcare sectors, and food industries [8,9,10,11,12].

In terms of physical characteristics, these polymers demonstrate gelation in the presence of inorganic salts. However, the gelation of these natural polymers can be controlled or improved, to display enhanced physicomechanical properties for viscosupplementation therapy. Synovial fluids are viscous, non-Newtonian, and are found in the cavities of synovial joints with high concentrations of protein. In synovial fluids, hyaluronic acid (HA) and proteins play a key role for boundary lubrication. Several artificial synovial fluids, i.e., hyaluronan and glucosamine, have been reported for joint injection therapy [13]. Some amphiphilic derivatives of alginate and hyaluronate, using long alkyl chains, i.e., dodecyl and octadecyl, have already been reported for cartilage repair [14]. In addition, hyaluronic acid derivatives have been reported as one of the current treatments for knee osteoarthritis, to replenish favorable viscosupplementation [15,16]. Furthermore, certain glycosaminoglycan derivatives have been reported for application as synthetic lubricants, possessing thixotropic properties [17].

Alginate derivatives have become an extremely important polysaccharide due to their potential applications in the field of biomedical applications [18,19,20,21,22,23]. Sodium alginate has been extensively used as a drug delivery carrier for application in tissue engineering, due to its biocompatible nature, low toxicity and easy gelation properties with divalent cations e.g., Ca^2+^ [24]. Conventional uses of alginate as an excipient in drug products depend on various properties, such as gel thickening, gel formation, and bio-stability [25]. This research demonstrates the functionalization of sodium alginate by 4-aminosalicylic acid (4-ASA) via amide bond formation. Several reports have indicated that gelling properties of sodium alginate can be induced via amide formation by adding different amine substrates on its backbone chain [26,27,28].

In the current study, we developed a novel microporous hydrogel based on sodium alginate and 4-ASA via carbodiimide coupling chemistry. This is the first report on the synthesis of an alginate-4-aminosalicylic acid based amide conjugate to the best of our knowledge. Furthermore, the modified hydrogel was found to be nontoxic and possesed drug release profiles simmilar to sodium alginate. Gelling ability and gel structure deformation/recovery behaviour was confirmed using rheological studies to evaluate the hydrogel as a stress-triggered hydrogel system. Development and evaluation of biopolymer-based soft hydrogel systems provide great possibilities for designing smart synovial fluids and may act as a biomimetic aqueous lubricant for arthritis treatment. Moreover, the combination of thixotropy, nontoxicity and drug release capabilities of the hydrogel enables significant potential for clinical application such as viscosupplementation in arthritis intervention.

## 2. Results Discussion

### 2.1. Physicochemical Properties of Alg-4-ASA Conjugate

The Alg-4-ASA amide conjugate was synthesized using varying ratios of Alg: 4-ASA. Yields of the different amide conjugates at various molar ratios 1:0.5, 1:1, and 1:2 were 59%, 66%, and 73%, respectively (Table 1). The amide conjugate under optimized conditions was used for further characterizations. The formulation was evaluated by FTIR and NMR spectroscopy. An optimized yield of 66% *w*/*w* was obtained with a molar ratio of 1:1, which exhibited favourable responses to external stimuli during gel to sol inter-conversion. It was further observed that formulation with a 1:2 molar ratio was not soluble in water. The conjugate with molar ratio 1:1 was, thus, employed for further studies and specific characterizations.

It should be noted that the amide conjugates were dispersed homogenously using ultrasonication in ambient conditions (Frequency 20 KHz for 5 min at temperature 60 °C followed by cooling at 20 °C). Alg (3% *w*/*v*) demonstrated dynamic shear viscosity of 339.22 mPas on the applied shear rate 100 S^−1^ at 37 °C, while amide derivatives Alg-4-ASA (1:0.5) and Alg-4-ASA (1:1) under identical experimental conditions exhibited 1040.2 and 8095.3 mPas, respectively (Table 1). The change of shear viscosity was observed due to numerous possible inter- and intra-molecular hydrogen bonding interactions, caused by insertion of 4-ASA moiety onto the polymer backbone. Intermolecular interactions between –COOH of 4-ASA side chains with –COOH of alginate back bone, as well as –COOH of 4-ASA side chains with –OH of alginate back bone was observed. Intramolecular interactions of –CONH group with –OH of Alg, –COOH of the non-substituted ring with –OH of the same ring and bonding between hydroxyl groups of adjacent sugar units were also observed.

### 2.2. Spectroscopic Characterization of Alg-4-ASA Derivatives

**Figure 1:**
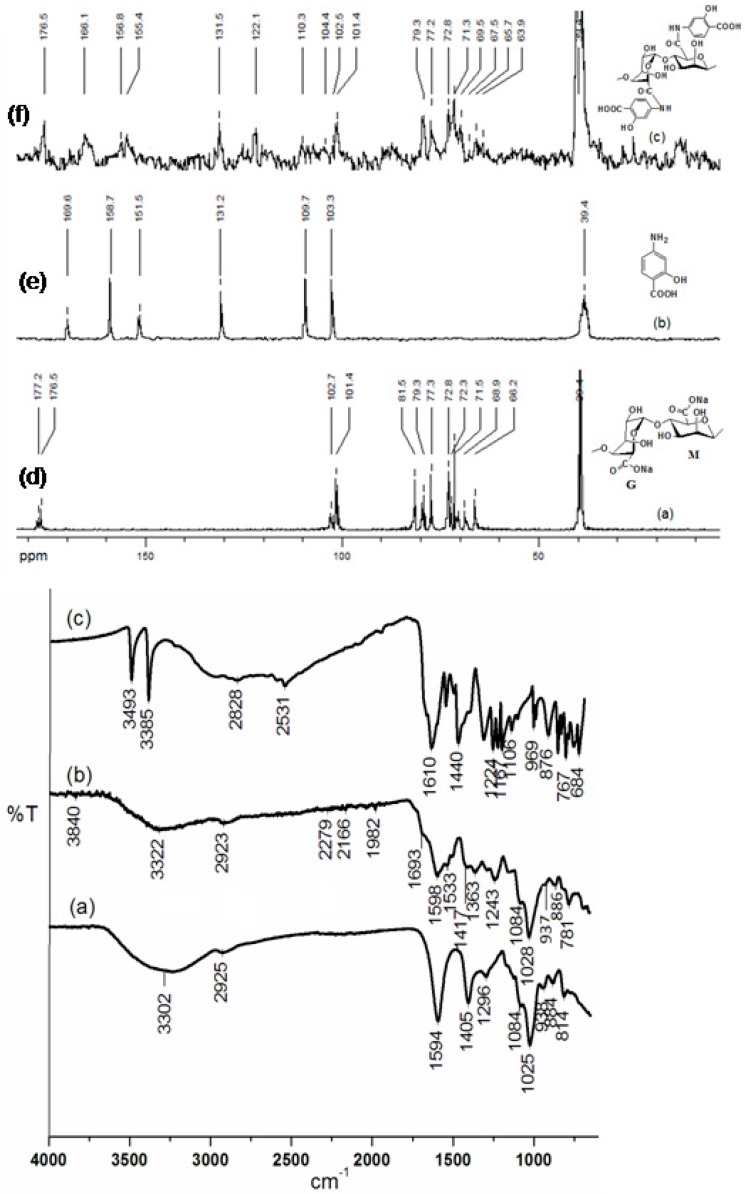
FTIR spectra of (**a**) Alg, (**b**) Alg-4-ASA (1:1), (**c**) 4-ASA; ^13^C-NMR spectra of (**d**) Alg, (**e**) 4-ASA, and (**f**) Alg-4-ASA (1:1).

FTIR and C^13^ NMR spectroscopy were undertaken for further confirmation of amide bond formation in the conjugates. In the FTIR spectral analysis, alginate exhibited its characteristic IR bands, as follows: (Figure 1a): ν_max_ (cm^−1^) 3302 br (–OH stretch), 2925 w (CH stretch), 1594 s (–CO stretch), 1405 w (C-C bend), 1084 and1025 s br (C–O–C stretch), and 884 cm^−1^ (C_1_–H β-mannuronate unit) [29,30,31]. The FTIR spectrum of amide conjugate in Alg-4-ASA (1:1) was shown in Figure 1b, which exhibited ν_max_ (cm^−1^) at 3304 s (–OH and/or NH stretch), 2923 m (CH_2_ and CH stretch), a band of the carbonyl (CO) stretch for amide (amide I band) at 1693 s cm^−1^, 1598 m (–NH, secondary amide II band), and 1243 m (–NH, amide III band) [29,30,31,32]. Other bands of the derivatives were in line with those of pure alginate, as well as 4-ASA (Figure 1c).

The ^13^C-NMR spectra of Alg, 4-ASA, and Alg-4-ASA (1:0.5) derivatives are shown in Figure 1d–f. The signal assessment for each spectrum is given in Table 2. The δ values for the NMR spectrum of Alg were similar to those reported in the literature (Figure 1d) [27]. Seven carbons of 4-ASA appeared at 169.6 ppm, 158.7 ppm, 151.5 ppm, 131.2 ppm, 109.7 ppm, and 103.3 ppm (C-1′ to C-6′) (Figure 1e). ^13^C-NMR spectrum of Alg-4-ASA (1:1) confirmed the covalent bonding of 4-ASA and alginate via amide bond formation. The conjugate exhibited peaks at 176.5 ppm and 166.1 ppm, which shifted up-field from 177.2 ppm (C_6_ carbon of guluronate) to 176.5 ppm (C_6_ carbon of guluronate), respectively (Figure 1f). This is potentially due to a shielding effect of the aromatic moiety and the inductive (–I) effect of the nitrogen atom (at C_6_ of uronate units) in the derivative.

However, several new peaks in the range of 101 ppm to 157 ppm for C1–C6 of the 4-ASA moiety confirmed insertion of this aromatic moiety into the polymer backbone via amide bond formation (Figure 1f). Other NMR signals in the spectrum of Alg-4-ASA (1:1) were identical to that of the reference polymer, i.e., sodium alginate. All the appeared carbon resonances correlated with those reported in previous literature [27].

### 2.3. Powder X-ray Diffraction Evaluation

**Figure 2:**
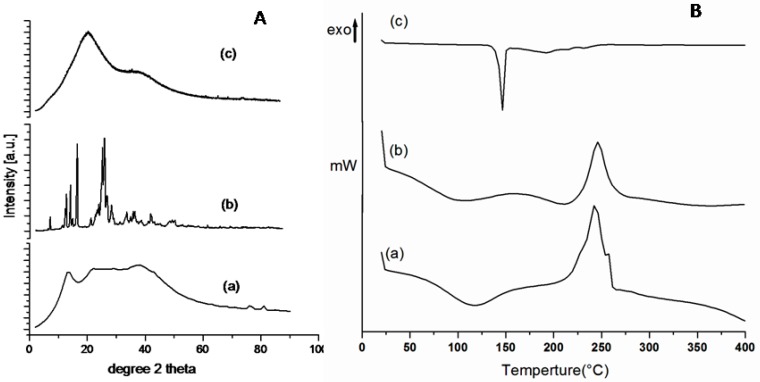
(**A**) Powder X-ray diffraction study of (**a**) Alg, (**b**) 4-ASA and (**c**) Alg-4-ASA (1:1); and (**B**) DSC analysis of (**a**) Alg (**b**) Alg-4-ASA (1:1), and (**c**) 4-ASA.

X-ray diffraction patterns of Alg-4-ASA (1:1) amide, along with the parent polymer sodium alginate and 4-ASA, are depicted in Figure 2A. Alg-ASA amide conjugates exhibited a broad peak at ~21° which was much sharper (Figure 2(Ac)) compared to that of sodium alginate at the same degree (Figure 2(Aa)). This varying pattern of broad peak indicates the amorphous nature of this amide conjugate even after the introduction of the 4-ASA moiety to the polymer backbone. In addition, the crystalline peaks at ~17° and ~26° in the X-ray pattern of pure 4-ASA powder (Figure 2(Ab)) also supports the sharper broad peak which appeared at 21° in the amide conjugate. Thus, the X-ray pattern of the amide conjugate demonstrates the amorphous morphology of the conjugated formulation.

### 2.4. Thermal Analysis of the Alg-4-ASA Conjugate

Thermal behaviour of the synthesized conjugate was analysed using DSC. Thermographs demonstrated the heating rate influence on the molecular weight of all conjugates against its native control polymer under N_2_ atmospheric conditions (Figure 2B). Amide conjugate Alg-4-ASA (1:1) demonstrated multiple endothermic peaks. The first endothermic peak was observed at approximately 100 °C, suggesting the dehydration process. An additional endothermic peaks close to 200 °C and 275 °C (Figure 2(Bb)) which was not seen in the DSC analysis of sodium alginate (Figure 2(Ba)), confirmed the different phase transitions on the conjugate which lead to the formation of an amide derivative. An exothermic peak at 350 °C in the conjugated gel was attributed to the decomposition of the polymer, indicating almost 100 °C greater than that of the parent polymer sodium alginate, displayed at 250 °C (Figure 2(Ba)). These results also provide a better thermal stability to the synthesized amide conjugate. In addition, there is a sharp endothermic peak near 150 °C, representing the melting of 4-ASA (Figure 2(Bc)) which was not seen in that of the amide conjugate, confirming the covalent bond formation instead of the physical mixture. The appearance of additional endothermic peaks in Alg-4-ASA confirms the 4-ASA moiety to the polymer backbone. Additional endothermic peaks in the Alg-4-ASA between 200–275 °C also indicate the changed thermal property of this conjugate, which may be attributed to its physical property, e.g., sol/gel transition, viscosity, etc.

### 2.5. Ultrasonication Induced Gelation of Alg-4-ASA Conjugates

Ultrasonication is a well-known method widely used for gelation and dispersion of several macromolecules. Herein, amide conjugates with mole ratio 1:0.5 and 1:1 were homogenously dispersed in water using an ultrasonicating probe (frequency 20 KHz for 5 min at temperature 60 °C), followed by cooling at 20 °C. The resultant suspension was observed in the form of a stable gel. Though gelation of conjugates were controlled and tuned using sonicating strength and the concentration of the derivatives, the higher the Alg-4-ASA concentrations, the faster the gelation observed. This study revealed that the ultrasound-induced formation of the gel provides a speedy and simple method for gel formation. Both the amide conjugates include numerous amide and additional hydroxyl functional groups, with molar ratio influences through inter-molecular hydrogen interactions, for resulting gelation. In addition, gelation of both amide conjugates were observed under sonication followed by cooling, possibly due to the molecular size of the Alg-4-ASA, being large enough to interact with the water molecules via formation of non-covalent bonds. Moreover, the elastic property of the gels, due to the 4-ASA moiety, resulted in intermolecular hydrogen bonding. These hydrogen bonds, thus, create a crosslinked supramolecular network, trapping water inside particle cavities, further contributing to the elastic nature of the material. In the case of alkaline medium, deprotonation of these carboxylates inhibits the above phenomena [33]. It was further noted that the Alg-4-ASA conjugate (ratio 1:1), exhibited strong gelation using sonication, compared to the other amide derivative (ratio 1:0.5). The reason for the weak gelation of the latter derivative Alg-4-ASA (1:0.5) could possibly be due to the lower amide and acid functional group reactivity.

### 2.6. Stress Responsiveness of Alg-4-ASA Hydrogel by Rheological Evaluation

**Figure 3:**
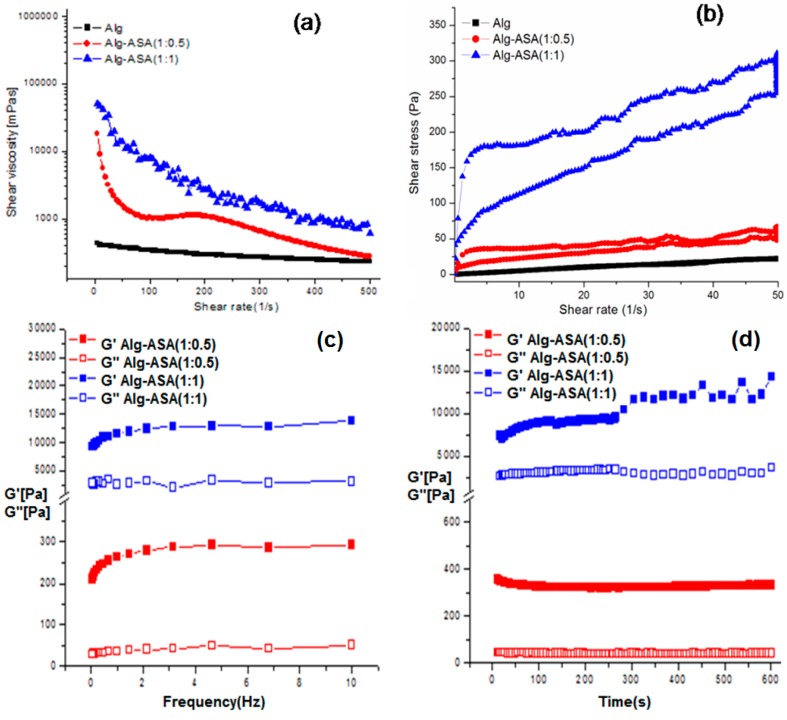

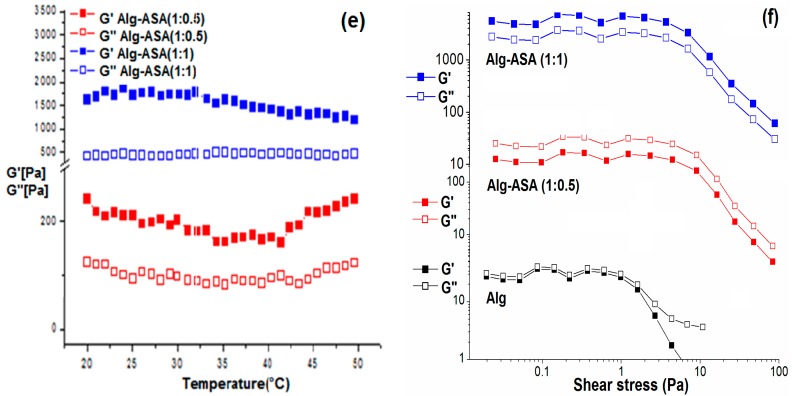
Rheological studies of Alg-4-ASA conjugate gels: (**a**) shear viscosity, (**b**) hysteresis loop area experiment, (**c**) oscillation frequency sweep experiment, (**d**) time ramp sweep experiment, (**e**) temperature ramp sweep experiment, and (**f**) oscillatory stress sweeps studies of hydrogel against the control polymer (concentration of each sample was 3% (*w*/*v*), pH (5–6)). All rheological analyses were carried out at 37 °C.

The ultrasound-induced gels, as discussed above, were stress switchable, being stable at room temperature without any external stimuli. On applied shearing, these gels were readily converted to the original solutions. Furthermore, solutions could form gel again upon removal of applied stimuli, demonstrating its reversible flow behaviour. The described sol–gel phase transition, for all these conjugates can be repeated several times with continued display of stimuli-responsiveness. However, the Alg-4-ASA (1:1) conjugate exhibited an enhanced response to applied stimuli, compared to the Alg-4-ASA (1:0.5). Shear viscosity of Alg-4-ASA conjugate gels and the control polymer are presented in Figure 3a. Gradual decrements in the shear viscosity (*η*) with increasing shear rate (*γ*), indicated a shear thinning property for all solutions, which is one of the desired property of a material to be used for viscosupplementation. It was noticed that the viscosity of Alg-4-ASA (1:1) was much higher at every shear rate compared to the Alg-4-ASA (1:0.5), as well as the native polymer. The higher viscosity of the Alg-4-ASA (1:1) indicated the formation of a stronger gel network due to the hydrogen bond interactions between the conjugate and water. Furthermore, both amide conjugate gels were found to have definite hysteresis loop area (Table 1) upon applied shear rate (Figure 3b), indicating their thixotropic nature. The observed thixotropy for amide conjugates, thus demonstrates their potential use as viscosupplementation (injectable gels) in biomedical applications. 

Further assessment of stress responsiveness for these materials using frequency sweep technique can be seen in Figure 3c. The storage and loss modulus were measured as a function of frequency at 37 °C. The results revealed that the storage modulus (G′) was observed to be greater than loss modulus (G′′) at all applied frequencies for both amide conjugate gels. However, the values for G′ of Alg-4-ASA (1:1) were higher than those of Alg-4-ASA (1:0.5), confirming stronger gel network strength in higher concentrated conjugates. This behaviour is typical for a standard hydrogel, which indicates an intermediate stage for the material between liquid and solid states [34]. This characteristic of the hydrogel further supports its potential application as viscosupplementation.

In addition, the thixotropic property of the gels Alg-4-ASA (1:0.5) and Alg-4-ASA (1:1) were observed. Storage and loss modulus as a function of time (0–600 s) at controlled shear (1 s^−1^) and temperature (37 °C) was carried out (Figure 3d). The experimental data demonstrated typical viscoelastic type behaviour of the gel material with less time dependency, where storage modulus (G′) of both conjugates was predominant over loss modulus (G′′) throughout the experiment, indicating more solid like behaviour of the gel [35]. In addition, stable values for G′ and G′′ throughout the experiments demonstrated colloidal stability of the gels [36]. In the case of the Alg, elastic modulus was not observed over all frequencies and time ranges, indicating very weak gel/solution nature of the native polymer. The frequency and time independent storage modulus (G′) was significantly higher than loss modulus (G′′) for amide conjugate gels, supporting the formation of their strong gel networks [37,38].

In the temperature sweep experiment, storage modulus (G′) and loss modulus (G′′) remained constant, with increasing temperature (20–50 °C), reflecting their thermal stability over this temperature range (Figure 3e). A minor decrement in storage modulus of the gel Alg-4-ASA (1:0.5) was shown above 30 °C, most likely due to higher mobility of the conjugate [39]. Rheological studies of the conjugate gels, thus, revealed shear responsiveness, significantly due to the 4-ASA moiety incorporation onto the polymer backbone.

Stiffness of the hydrogel was measured using an oscillatory stress sweep measurement technique (Figure 3f). From this experiment it can be seen that both Alg-ASA conjugates were much stiffer as it showed higher G′ values and greater the yield point (higher shear stress) compared to the individual polymers (alginate). These rheological results were in the same line of frequency sweep experiments, where the storage modulus (G′) were observed to be greater than loss modulus (G′′) at all applied frequencies for both amide conjugate gels.

### 2.7. Morphological and In Vitro Cytotoxicity of the Alg-4-ASA Conjugate

**Figure 4:**
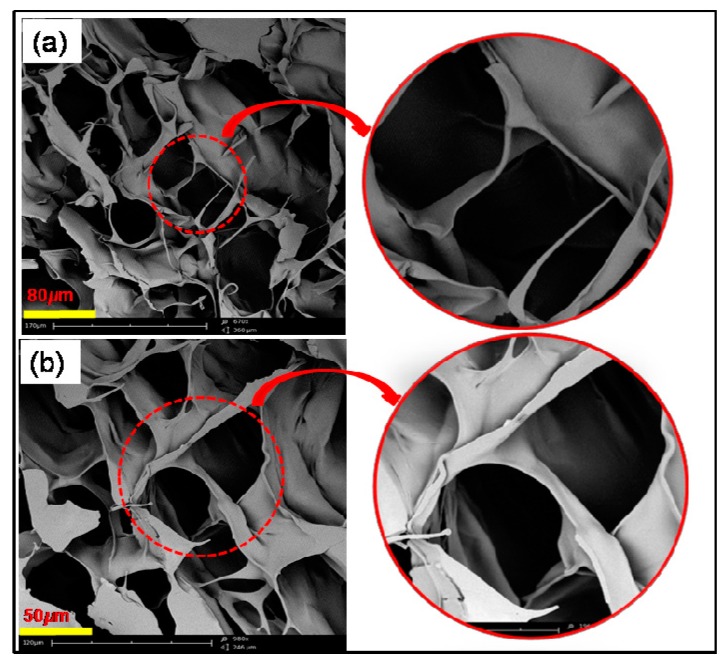
Scanning electron micrographs of hydrogels: (**a**) Alg-4-ASA (1:0.5) and (**b**) Alg-4-ASA (1:1).

Morphological analysis was carried out on the conjugated hydrogel. As observed in Figure 4a,b, uniform gel network arrangement and pore structure can be attributed to the thixotropic characteristics of the gel [40]. The gel structure of the samples further displayed a regular layered and highly porous configuration, thus responsible for the water retention property of the conjugated hydrogel. 

**Figure 5:**
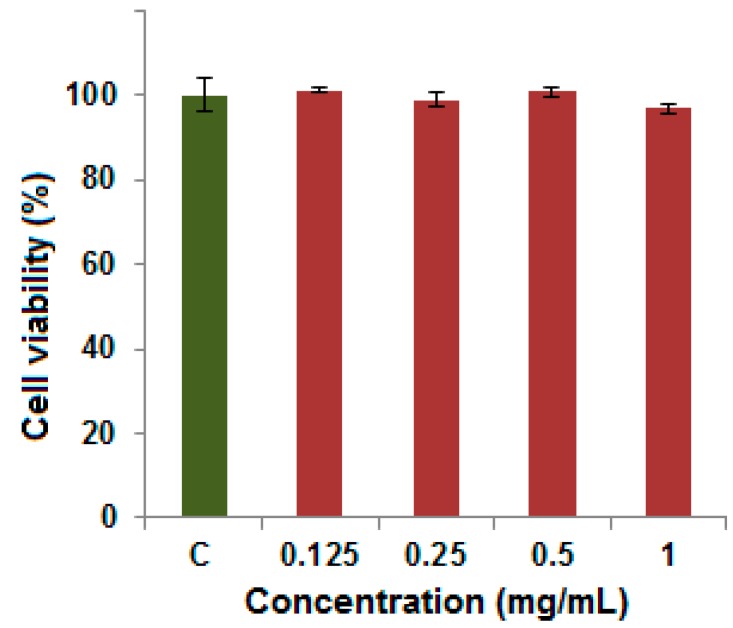
Cell viability of human dermal fibroblast-adult cells incubated with different concentrations of Alg-4-ASA (1:1) hydrogel.

The use of materials for producing a viscosupplementation formulation for arthritis treatment must be non-toxic for such application. To investigate the toxicity level of this material, a cell viability assay was carried out. Human dermal fibroblast-adult (HDFa) cells were incubated with different concentration of the microporous hydrogel for 24 h. The cell viability as shown in Figure 5 remained over 95% in the range of 0.125–1.0 mg/mL, indicating good cellular biocompatibility for clinical application.

### 2.8. In Vitro Drug Release Evaluation

**Figure 6:**
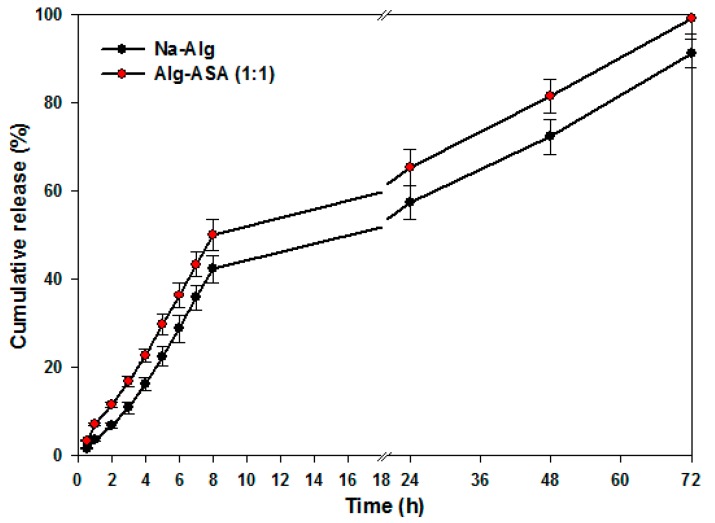
The release profiles of aspirin loaded hydrogels within 72 h in PBS (pH = 7.4) at 37 °C.

In vitro drug release using Alg-4-ASA hydrogel as a carrier was studied at 37 °C, using aspirin as a model drug. As observed under SEM analysis, drug can be easily incorporated within the microporous structure of the hydrogel. To illustrate whether the introduction of 4-ASA moiety to the sodium alginate backbone affected the drug release performance of the native polymer, evaluation of aspirin-loaded Alg-4-ASA and aspirin-loaded Alg were both evaluated in PBS (pH = 7.4), as seen in Figure 6.

In vitro results clearly indicated that no substantial difference between drug-release rates of the native polymer and the synthesized conjugate was observed. Aspirin concentrations of 42.8% and 49.6% within 8 h from alginate and Alg-4-ASA (1:1) hydrogels was observed, respectively. Furthermore, 90.1% and 97.6.5% of aspirin was released from alginate gel and Alg-4-ASA conjugate respectively within the following 64 h (8–72 h), suggesting sustained drug release patterns. The study demonstrated that the modified polymer still possesses good drug release performance similar to pure sodium alginate hydrogel.

A possible explanation for similar release profiles can be attributed to common chemical configuration properties of the planar structure of the native and conjugated hydrogel systems. Insertion of the 4-ASA aromatic moiety to the native polymer did not significantly affect the polymers drug release capacity, however, the thixotropic and lubrication properties of the conjugated system was substantially enhanced. The drug release profile of the conjugated amide thus indicates that the designed hydrogel could be used as an intelligent drug carrier system, triggered by external stimuli like physical stress, which can be applicable when an immediate high dosage is required for instant relief.

## 3. Materials and Methods

### 3.1. Materials

Alginic acid sodium salt (Na-Alg, Mw: 75 kDa; M/G ratio: 0.6), 4-aminosalicylic acid (4-ASA), *N*-(3-dimethylaminopropyl)-*N*′-ethylcarbodiimide hydrochloride (EDC), 2-(*N*-Morpholino) ethane sulfonic acid (MES) buffer, and *N*-Hydroxysuccinimide (NHS) were of analytical reagent grade and was purchased from Sigma Aldrich^®^, Johannesburg, South Africa. All other chemicals used were of the highest percentage purity and were used as procured.

### 3.2. Synthesis of Sodium Alginate-4-Aminosalicylic Acid Amide Derivative (Alg-4-ASA)

For the preparation of the amide conjugate using EDC as a coupling agent, the synthetic process outlined by Chejara and co-workers was followed [27]. Briefly, 4-ASA (0.04–0.23 g) was added to 20 mL of sodium alginate solution (1% *w*/*v*), which was prepared in 0.1 M MES buffer (pH 6.0). The reaction mass was allowed to agitate for 10 min at room temperature for homogeneous dispersion of all reagents. Further 0.287 g (2.5 mmol) of NHS and 0.787 g (4.12 mmol) of EDC were added to the reaction mixture and allowed to stir for 12 h at 25 °C. After completion, the formulation was dialyzed in water using a dialysis membrane (MWCO: 1.2 kDa) to remove unreacted ingredients. Finally, the resulting purified formulation was lyophilized to obtain the modified sodium alginate-4-ASA amide conjugate.

### 3.3. Spectral Characterization of the Synthesized Amide Conjugate

Alg-4-ASA amide conjugate was characterized using FTIR, ^13^C-NMR, and powder X-ray diffraction analysis. FTIR spectra for all samples were recorded on a Perkin Elmer Spectrum 2000 FTIR spectrometer, employing a single reflection diamond MIRTGS detector (PerkinElmer Spectrum 100, Liantrisant, Wales, UK). ^13^C-NMR spectra were recorded using Bruker Advance-II 500 spectrometer, Fällanden, Switzerland. Samples were prepared by dissolving them in D_2_O (30–40 mg/mL), and spectrum for Alg-4-ASA was recorded at 70 °C. Sodium alginate and the 4-ASA spectra were recorded at room temperature (25 °C) using *d*_6_-DMSO (ca. 39.4 ppm) as the standard. 4-ASA was dissolved in *d*_6_-DMSO (15–20 mg/mL). Powder XRD characteristics of the samples were analysed employing Rigaku MiniFlex600 Benchtop X-ray Diffractometer (Rigaku Corporation, Tokyo, Japan), fitted with a 600 W (40 KV–15 mA) X-ray generator and a high intensity D/texultra-high speed 1D detector.

### 3.4. Thermal Analysis of the Synthesized Amide Conjugate

Thermal characteristics of the synthesized amide conjugate and individual polymers were determined using differential scanning calorimetry (DSC) analysis (DSC 1, STAR^e^ Mettler Toledo, Schwerzenback, Switzerland). Approximately, 10 mg of each sample was sealed into aluminum pans for analysis and were gradually heated (heating rate: 10 °C/min) in the range of 20 °C to 400 °C.

### 3.5. Preparation of the Microporous Hydrogel

Alg-4-ASA amide conjugate was homogenously dispersed in distilled water using 3.0% *w*/*v* concentration via ultrasonication technique (pulse: Amp 1, 80% for 30 s) at 60 °C, followed by cooling the formulation to 20 °C for the preparation of the microporous hydrogel. Ultrasonication was applied using SONICS Vibra cell (Model CV18; 130 Watt at 20 KHz).

### 3.6. Surface Morphological and In Vitro Cytotoxicity Evaluation of the Microporous Hydrogel

Surface morphology and microstructure analysis of the synthesized amide conjugate was studied using scanning electron microscopy (SEM), which was performed employing a Phenom^TM^ SEM (FEI Company, Hillsboro, OR, USA). Lyophilized hydrogel samples were mounted on a sample holder and coated with a gold isotope for 60 s, using a gold-sputter coater (SPI Module^TM^ Sputter Coater, Structure Probe, Inc., West Chester, PA, USA) prior to SEM imaging.

In vitro cytotoxicity of Alg-4-ASA amide hydrogel was evaluated by standard MTT assay using Human Dermal Fibroblast-adult (HDFa) cells. These cells were seeded with density of approximately 104 cells/well on plates (96-well) and cultured for 24 h using standard cell culture media. The cultured cells were exposed to Alg-4-ASA amide hydrogel with different concentrations and incubated for 24 h, followed by removal of supernatants. Further PBS solution was used for washing of cells and 50 μL of MTT solution (0.5 mg/mL) was added to each well. After incubation for 4 h, the culture medium was removed and DMSO (100 μL) was added to the wells to dissolve precipitates. The absorbance of these samples were determined at 570 nm using a microplate reader. Cell viability was calculated using Equation (1):

Cell viability (%) = OD_treated_/OD_control_ × 100 (1)

where OD_treated_ was obtained from the cells treated with Alg-4-ASA amide, and OD_control_ was obtained from the untreated cells and defined as 100% viability.

### 3.7. Rheological Evaluation of Modified Hydrogel

Rheological analysis was undertaken employing a Haake Mars (II) Modular Advanced Rheometer system (Thermo Fisher SCIENTIFIC, Johannesburg, South Africa), using a cone plate geometry (Rotor C35/1, D = mm, 1° Titan) with a gap measurement of 0.050 mm. Hydrogel for alg-4-ASA (3% *w*/*v*) and sodium alginate (3% *w*/*v*) were homogenized before measuring their rheological properties. The following types of rheological experiments were performed: (a) shear viscosities with different shear rates; (b) frequency sweep experimentation; (c) time ramp sweep experimentation; and (d) temperature sweep experimentation. The hydrogels were also studied for their thixotropic properties using hysteresis loop area measurement technique. Shear viscosities of all samples at varying shear rates were determined at 37 °C. Oscillatory frequency sweeps for all samples were performed from 10 to 0.10 Hz at a constant shear stress of 1 Pa. Time ramp sweep experiment was studied up to 600 s to evaluate the variation in the storage and loss modulus of the hydrogel. Temperature ramp sweep experiment was performed to determine the thermal stability and thermal characteristics of the hydrogels. Oscillatory stress sweeps were performed by applying increasing shear stress logarithmically from 0.05 Pa 100 Pa at a fix frequency of 1 Hz. For evaluation of inherent thixotropic properties, samples were analyzed using hysteresis loop area technique, applying varying shear rates in the range of 0.1 to 50 s^−1^ for 600 s. All measurements were undertaken in triplicate. Hysteresis loop, thixotropic area and percentage thixotropic area (*Ar*) were calculated and obtained as described in literature [28].

### 3.8. In Vitro Drug Release Evaluation

In vitro release profiles of aspirin loaded Alg-4-ASA at different time periods was monitored up to 72 h, employing a dialysis tube diffusion method [41]. Alg-4-ASA conjugate which was loaded with aspirin, was enveloped into a dialysis membrane tube (Mw cut-off 10,000 Da). The dialysis membrane was dialyzed in phosphate buffer solution (500 mL; pH = 7.4) at 37 °C. At definite time intervals, 4 mL of the released buffer solution/medium was withdrawn and replaced with freshly prepared PBS buffer, to maintain constant volume. The absorbance was measured for the solution and the released amount of aspirin was determined by UV spectroscopy. The percent of aspirin released was calculated using Equation (2):

Drug release (%) = *M_t_*/*M_a_* × 100 (2)

where *M_t_* is the amount of aspirin released from the hydrogel sample at a definite time *t* and *M_a_* is the total encapsulated amount of aspirin in the Alg-4-ASA gel. Release studies were continued until no significant change was observed in concentration of the released drug. Each measurement was repeated three times and the average value was calculated.

## 4. Conclusions

A novel stress responsive microporous hydrogel system based on biopolymer sodium alginate, synthesized using carbodiimide coupling chemistry was undertaken. The microporous hydrogel demonstrated rapid gelation in the presence of ultrasonication and external stress, which was confirmed using various rheological techniques. Moreover, the designed microporous hydrogel exhibited good cell viability potential as well as similar drug release behaviour to that of its native polymer, following chemical modification. This approach of modification of biopolymers for application of specialized functional properties, has various benefits for designing innovative drug delivery systems. The developed conjugated stress responsive hydrogel system, thus, has immense potential in the field of biomedical applications, particularly for the treatment of joint injury and arthritis induced conditions.

## Figures and Tables

**Table 1:** Experimental data generated from rheological studies of native and conjugate hydrogels.

Samples *^a^*	Yield (% *w*/*w*)	Shear Viscosity (mPas) *^b^*	Thixotropic Area, Ar (%) *^c^*
Na-Alg	NA	339.22	0.59
Alg-ASA (1:0.5)	59.0	1040.20	24.83
Alg-ASA (1:1)	66.0	8095.30	26.23
Alg-ASA (1:2)	73.0	NA	NA

*^a^* Concentration of sample were 3% (*w*/*v*), at pH 5–6 at 37 °C; *^b^* Shear viscosity is reported from shear viscosity vs. shear rate (shear rate 100 s^−1^); *^c^* Data presented values are the average of three measurements.

**Table 2:** ^13^C-NMR chemical shifts (ppm) observed for non-modified Alg, 4-ASA, and Alg-4-ASA (1:1) amide conjugate.

Assignment (δ ppm)	Na-Alg	4-ASA	Alg-ASA (1:1)
C-1 MM	102.7	NA	102.5
C-2 MM	72.3	NA	72.7
C-3 MM	72.8	NA	71.3
C-4 MM	79.3	NA	77.2
C-5 MM	77.3	NA	72.8
C-6 MM	176.5	NA	176.5
C-1 GG	101.4	NA	101.4
C-2 GG	66.2	NA	63.9
C-3 GG	71.5	NA	67.5
C-4 GG	81.5	NA	79.3
C-5 GG	68.9	NA	65.7
C-6 GG	177.2	NA	176.5
C-1′	NA	109.7	110.3
C-2′	NA	131.2	131.5
C-3′	NA	109.7	110.3
C-4′	NA	151.5	155.4
C-5′	NA	103.3	104.4
C-6′	NA	158.7	156.8
C-7′	NA	169.6	166.1

NA = Not applicable.
